# Exploring Morphological, Transcriptomic, and Metabolomic Differences Between Two Sister Lines with Contrasting Resistance to Orange Rust Disease in Sugarcane

**DOI:** 10.3390/ijms26083490

**Published:** 2025-04-08

**Authors:** Yupeng Zhou, Edvin Sebastian Mihail, Ziliang Luo, Sushma Sood, Md Sariful Islam, Jianping Wang

**Affiliations:** 1 Department of Agronomy, College of Agricultural and Life Sciences, University of Florida, Gainesville, FL 32611, USA; yupeng.zhou@wur.nl (Y.Z.); e.mihail@ufl.edu (E.S.M.); 2 Sugarcane Production Research Unit, USDA ARS SEA, 12990 US Hwy 441 N, Canal Point, FL 33438, USA; sushma.sood@usda.gov

**Keywords:** metabolomics, orange rust, *Pucinnia kuehnii*, sugarcane, transcriptome

## Abstract

Sugarcane (*Saccharum* spp.) hybrid, one of the most important crops in Florida, has been affected by orange rust (OR) disease caused by *Puccinia kuehnii* since 2007, resulting in significant yield loss. Developing resistant cultivars to this disease has become an important goal in sugarcane breeding programs. However, the specific genes and molecular mechanisms underlying the resistance to OR disease in sugarcane are still not clear. In this study, we selected two sugarcane sister lines with different genotypes—showing contrasting resistance responses to the disease—from a major quantitative trait loci (QTL) region controlling OR disease resistance. Morphological and anatomical observations revealed that the resistant line (540) had significantly smaller stomatal size and lower stomatal density than the susceptible line (664). Transcriptomic analyses showed that resistant line 540 had increased cell surface modification activity, suggesting possible increased surface receptors. Differentially expressed gene and coexpression analyses also revealed key genes involved in the biosynthesis of anti-fungal molecules, such as hordatines, arabidopyrones, and alkaloids. They also showed a strong increase in long non-coding RNA expression, playing a role in transcriptional regulation. Transcriptomic–metabolomic joint analysis suggested that the biosynthesis of phenylpropanoid derivatives with purported antioxidant and anti-fungal capabilities increased in line 540, especially those deriving from ferulate. Genes, pathways, and some single-nucleotide polymorphisms identified in this study will provide fundamental information and resources to advance the knowledge of sugarcane molecular genetic mechanisms in relation to OR disease, supporting breeding programs in developing cultivars with improved resistance to OR.

## 1. Introduction

Sugarcane (*Saccharum* spp.) hybrid, as a perennial C4 crop, is broadly grown in tropical and subtropical areas in the world, contributing significantly to global sugar production [1,2,3]. In the U.S., Florida is one of the leading states in sugarcane production [4,5]. The sugarcane industry is economically significant, generating over USD 1 billion annually [6].

Part of the Poaceae family, sugarcane includes six species: two wild species, *S. robustum* (2n = 10x = 60–170) and *S. spontaneum* (2n = 8x = 36–128), and four cultivated species, *S. officinarum* (2n = 8x = 80), *S. barberi* (2n = 81–124), *S. sinense* (2n = 111–120), and *S. edule* (2n = 60, 70, 80) [1,7,8]. Modern sugarcane cultivars are derived from interspecific crosses between *S. officinarum* and *S. spontaneum*, followed by repeated backcrosses with *S. officinarum*. As a result, modern sugarcane cultivars have up to 130 chromosomes, primarily from *S. officinarum* (~80%), much less from *S. spontaneum* (~10–20%), and a portion from recombination [1,7,9,10]. Due to high polyploid levels, every gene in a specific cultivar has multiple copies or alleles (10–14) [11,12,13,14]. The multiple alleles and gene copy numbers can make genome assembly and allele definition extremely challenging. This complexity has caused sugarcane genomics to lag behind other monocot crops like rice and maize [10]. Despite these challenges, Garsmeur et al. (2018) first sequenced and assembled a monoploid genome using a bacterial artificial chromosome (BAC)-based monoploid genome sequence of the French cultivar R570 [15]. In the meantime, the haploid S. *spontaneum* “AP85-441” (1n = 4x = 32) was assembled, although it contributes to only a small percentage of genomes in modern sugarcane cultivars [16]. Recently, reference genomes of two typical modern cultivars, R570 (approximately 2n = 12x ≈ 114) and Zhong Zhe No. 1 (ZZ1, approximately 2n = 12x = 114), were published, expanding the avenues for unraveling the complex polyploid genome [17,18]. The available reference genomes are critical foundations for molecular genetics studies of any trait in sugarcane.

Sugarcane orange rust disease (SORD) is caused by a biotrophic fungal pathogen, *Pucinnia kuehnii* E.J. Butler. It became a severe disease in 2000 in Australia, resulting in up to a 50% loss of production of the dominant variety Q124 and a loss of AUD 150–210 million [19,20]. In 2007, *P. kuehnii* was first detected in the Western Hemisphere, on the brown rust-resistant cultivar CP 80-1743 in Florida, causing a potential yield loss of up to 43% by reducing the sugarcane photosynthetic rate [21,22,23]. Other countries in North and South America also started to report orange rust in the following years [24]. As SORD has progressively spread across many countries, identifying resistance sources has become an important focus in many breeding programs. In the realm of plant immunity, certain morphological factors, such as stomata, cell wall, and cuticle thickness, can passively deter fungal pathogens. These factors may combine with active responses involving anti-fungal molecules to further protect against infection [25]. For example, previous research has shown that bacteria and fungi can induce stomatal closure, a process known as stomatal immunity, to prevent pathogen entry and proliferation [26,27]. However, there is limited research on the resistance of plants to orange rust in relation to plant morphology. Identifying resistance genes is the most efficient approach to guide future sugarcane breeding for resistant cultivar development. Yang et al. (2018) identified three major QTLs, qORR109, qORR4, and qORR102, accounting for 58%, 12%, and 8% phenotypical variation, respectively, in a bi-parental segregating population derived from a cross between a resistant sugarcane breeding line and a susceptible cultivar [24]. These SORD resistance QTLs are critical for identifying candidate genes to control SORD resistance and for developing markers to assist in the selection of resistant cultivars. Approaching this challenge by integrating genomics, metabolomics, and transcriptomics techniques significantly contributes to unraveling the genetic underpinnings of how plants respond and adapt to challenging biotic/abiotic conditions, while also aiding in the identification of crucial genes and pathways. Moreover, increasing evidence suggests that non-coding RNAs (ncRNAs) play important roles in plant immune responses [28]. However, there is limited research on using multi-omics in studying SORD. Through time-series transcriptome analysis of a susceptible genotype, Correr et al. (2020) revealed an initially suppressed defense system, or the mechanism of susceptibility in the susceptible genotype [7]. However, the exact mechanisms contributing to orange rust resistance are still largely unknown.

To further narrow down the genomic regions controlling orange rust resistance, particularly qORR109, it is important to have a large segregating population for fine mapping the candidate gene(s), which can be a challenge given the complex sugarcane genome. In this research, we selected two sister lines from the above segregation population for the QTL analysis [24]. The two sister lines had contrasting phenotypes in response to SORD and had different genotypes of the major QTL qORR109 [24]. One line showed resistance to SORD and the other showed susceptibility. The resistance line had a resistance genotype of the major QTL qORR109, while the susceptible one had a susceptible genotype, which probably due to prehaustorial resistance. Haustoria mother cell are seen approximately 12 hai (hours after the inoculation) and haustoria formed within 24 h [29]. We hypothesize that the morphological variations and differential transcriptomic and metabolomic responses between resistant and susceptible lines during the early infection stage led to their distinct disease resistance phenotypes. In this research we tested the hypothesis above by surveying the morphological variations and differential transcriptomic and metabolic responses between these two lines. So, the specific objectives of this study were to compare the two selected sister lines by (1) observing their basic plant leaf morphology, (2) exploring transcriptome profiling within 24 hai and (3) investigating metabolic profiling after SORD infection. A previous transcriptome study examined a susceptible sugarcane line responding to *Puccinia kuehnii* infection over a wide post-infection period. However, the plant’s reaction to *P. kuehnii* infection at earlier stages remains unclear, particularly given that plant defense and plant immunity could start early after the pathogen’s attack [30,31,32]. In addition, sugarcane’s metabolomic responses to SORD are rarely studied. Therefore, this study provides novel relevant information and resources regarding the resistance mechanisms governed by genes harbored in the major QTL, qORR109. The results will not only provide comprehensive resources and insightful information to facilitate future fine-mapping and map-based cloning of the underlying resistance genes when substantial segregating populations are established, but will also help reveal various factors contributing to SORD resistance.

## 2. Results

### 2.1. Morphological Observations

**Figure 1:**
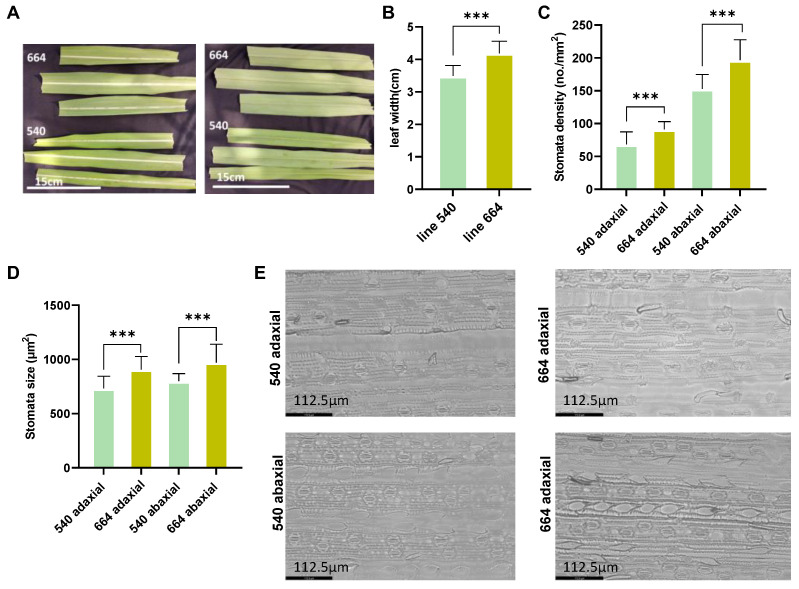
(**A**) Adaxial and abaxial surfaces of the two lines (resistant line 540 and susceptible line 664). (**B**) Leaf width of the two lines. (**C**) Stomata density of the two lines. (**D**) Stomata size of the two lines. (**E**) Microscopic observation of stomata of the two lines. Each bar in the figure includes the mean value along with the standard deviation. (*** *p* < 0.001).

Leaf width, leaf stomatal size, and stomatal density were observed to display morphological variations between two contrasting sister lines. The leaf width of the resistant line 540 was significantly (16.58%) narrower than that of the susceptible line 664 (Figure 1A,B). In addition, line 540 had a lower stomata density and smaller stomata size than line 664 on both the adaxial and abaxial sides of the leaf (Figure 1C–E).

The cross-sections of the leaves were further compared between the two lines. It was noticed that the leaves of line 540 seemed to have a condensed area occupied by the xylem and phloem, which may allow for better water and nutrient transport than line 664 (Figure S1). These results indicate that the morphological and physiological variations between the two sister lines may potentially contribute to their differences in orange rust resistance response.

### 2.2. mRNAseq Analysis

mRNA-seq analysis was performed on the two lines, 540 and 664, at five different time points (0, 3, 6, 12, and 24 HAI) with three biological replicates, thus producing a total of 30 samples for the experiment. In total, 2,066,179,518 raw 150 bp read pairs were obtained, with an average of 68,872,650.6 paired reads per sample, representing a sequencing depth of 52.8× of the sugarcane transcriptome, given the sugarcane transcriptome size of 391 Mb. After filtering and trimming, an average of 64,987,742.23 (94.36%) clean read pairs per sample was obtained. The clean read pairs were aligned to the *S. officinarum* genome with alignment rates ranging from 95.26% to 97.5%, with an average of 96.55%. The unaligned reads (on average, 4,438,365.77 reads per sample) against the *S. officinarum* genome were further aligned with the *S. spontaneum* genome [33]. About 30–50% of the reads were aligned to the *S. spontaneum* genome. However, no additional genes were identified.

**Figure 2:**
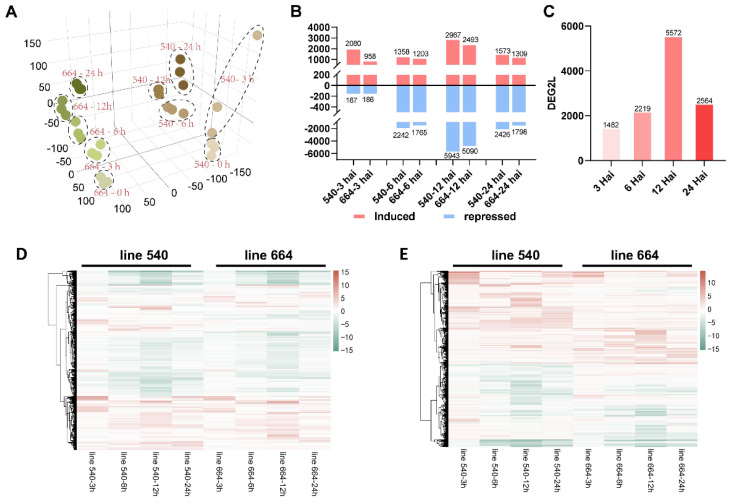
(**A**) Principal component analysis of the mRNA datasets. (**B**) Numbers of differentially expressed genes (DEGs) at different time points in two lines. (**C**) Numbers of differentially expressed genes between the two lines (DEG2Ls) at different time points. (**D**) Heatmap of log_2_ (fold change) of DEGs at different time points in two lines. (**E**) Heatmap of log_2_ (fold change) of DEG2Ls at different time points.

A total of 119,196 genes were actively expressed in the 30 samples of the two lines, accounting for approximately 47.06% of all sugarcane genes. Principal component analysis (PCA) of the 30 samples’ gene expression data showed that most of the three replicates of each line at every time point were closely clustered together. The two lines were well separated, suggesting consistent gene expression among the replicate samples and notable gene expression changes between the two lines and different time points (Figure 2A). Differentially expressed genes (DEGs) were identified by comparing the gene expression levels in samples of 3, 6, 12, and 24 hai, respectively, to 0 hai. As a result, a total of 17,812 non-redundant DEGs were identified in the two lines across all of the different time points. In general, more DEGs were identified in the resistant line 540 than in susceptible line 664 at any given time point (Figure 2B,D). The number of induced and repressed DEGs after SORD infection in both lines increased first and reached the highest level at 12 hai, and then decreased at 24 hai (Figure 2B,D), indicating that both lines were most actively responding to the infection at 12 hai compared to the other three time points in this study. Among the 17,812 DEGs, 13 DEGs were identified at all time points in the two lines combined (Table S1).

The expression level of all DEGs was further compared between lines 540 and 664 to identify the DEGs expressed significantly differently between the two lines at each time point, respectively, named as DEG2Ls (see Section 4.5), which showed more than three times difference in fold change between the two lines (Figure 2C,E). A total of 9406 non-redundant DEG2Ls were identified in 8 different sub-cases (Supplementary Table S2). The DEGs and DEG2Ls were distributed on all chromosomes, with more on Chr01, Chr02, Chr03, and Chr04 than on the other chromosomes (Table S3).

**Figure 3:**
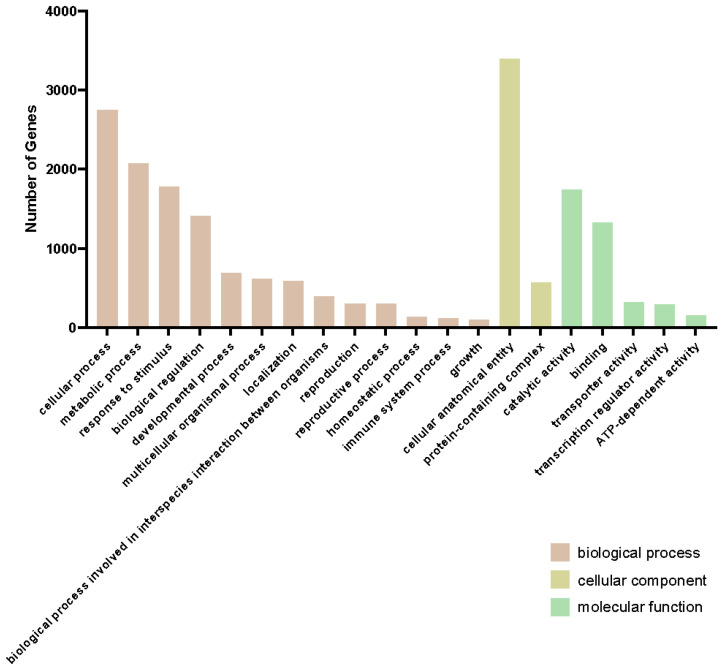
GO enrichment analysis of DEG2Ls. Top significantly enriched GO terms in the biological process category. Cellular components and molecular functions are shown for DEG2Ls. Bar plots represent the number of genes of different GO terms.

Most (84.97%) DEG2Ls were annotated, and about 42.78% were assigned with a Gene Ontology (GO) term. The GO enrichment of DEG2Ls was related to the response to stimulus, immune system process, different protein binding activities, and antioxidant activity, confirming our assumption that the resistant line had a quick immunity response at the transcription level to orange rust at a very early stage (Figure 3). The most enriched GO terms are “metabolic process”, “cellular anatomical entity”, and “catalytic activity”. Since these categories are abstract, we closely examined three DEG2Ls within these abstract GO terms and their functions and compared their expression in lines 664 and 540 for each category. In the “metabolic process”, phosphomethylpyrimidine (HMPP) synthase (O82392), for example, showed almost nine times higher expression at 12 hai in the resistant line 540 than in the susceptible line 664 (Figure S2A). Another example is thiamine thiazole synthase 2 (C5X2M4), which exhibited a much stronger expression response from 6 hai to 12 hai in line 540 compared to line 664 (Figure S2B). Copper-containing amine oxidase gamma 2 showed low basal expression in line 540, but peaked dramatically at 12 hai before returning to basal levels (Figure S2C). The GO term “cellular anatomical entity” also contained some interesting DEG2Ls. For example, 40S ribosomal protein S4-3 (Q8VYK6) started at a much higher expression level in the resistant line 540, but decreased over the course of infection; meanwhile, expression remained low in the susceptible line 664, suggesting line differences in protein synthesis (Figure S2D) during orange rust’s early infection. Photosystem II protein D1 (A1E9Q4) showed an increased expression level in line 664 over line 540 throughout infection from 12 hai (Figure S2E), which suggests how photosynthesis may be affected differently in the lines. As another example, aquaporin TIP4-1 (O82316) showed a similar expression level at 6 hai in both lines, but then showed a near two-fold difference in change at 12 hai, with line 540 having much higher levels (Figure S2F). The GO term “catalytic activity” was the most enriched in the “molecular function” class. Some interesting DEG2Ls in this category included agmatine coumaroyl transferase (Q9FNP9), which showed a spike in expression in line 540 at 3 hai, suggesting a very rapid response to SORD; line 664 also exhibited this spike, but at levels nearly 6 times lower in some replicates (Figure S2G). Thioredoxin-like 3-1 (Q9FG36) and putative calcium-transporting ATPase 11 (Q9M2L4) were also found in “catalytic activity”, and line 540 showed a near 7-fold higher expression level at 12 hai for both genes (Figure S2H,I). A good number of the DEG2Ls presented here show a peak expression at 12 hai before tapering off.

**Figure 4:**
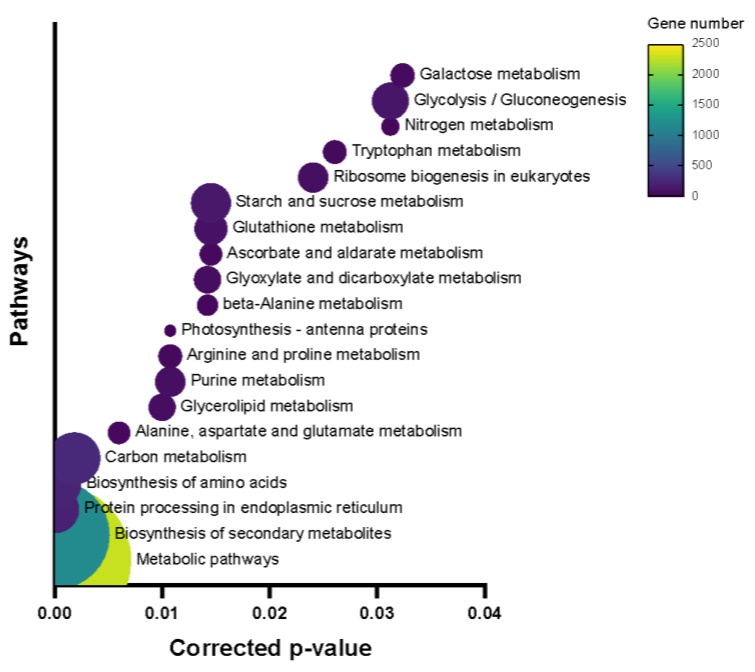
Top 20 metabolic pathways with the smallest *p*-values of the DEG2Ls identified by KEGG enrichment.

In order to have a better understanding of which pathways were highly involved after inoculation, pathway enrichment analysis was performed. This identified 19 pathways, with the pathways of “photosynthesis—antenna proteins”, “Monoterpenoid biosynthesis”, and “Limonene and pinene degradation” as the top three enriched pathways (Figure 4).

More importantly, the sequence of the G1 marker linked to the orange rust resistance contributed by the major QTL, qORR109 on chromosome 2 provided new insights to identify the essential genes responding to orange rust [24], and the G1 marker was mapped to the reference genome on chromosomes 2G, 2H, and 2F. Within a sequence window from 200 kb upstream to 200 kb downstream of the Chr2-G1 marker, 306 genes were obtained, out of which 7 genes (*Soffic.02G0012580-8H*, *Soffic.02G0012750-6H*, *LAp.02H0013130*, *LAp.02H0013490*, *Soffic.02G0005030-6G*, *LAp.02F0013720*, and *Soffic.02G0014380-5F*) were defined as DEG2Ls.

### 2.3. Long Non-Coding RNA (lncRNA) Analysis

**Figure 5:**
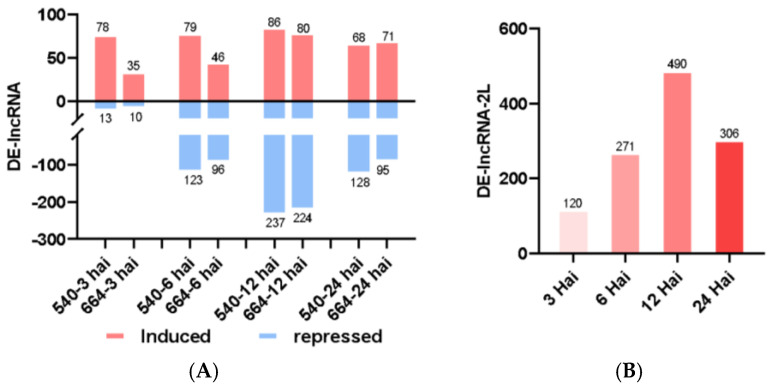
(**A**) Numbers of differentially expressed lncRNAs (DE-lncRNAs) at different time points in the two lines (resistant line 540 and susceptible line 664). (**B**) Numbers of DE-lncRNAs differentially expressed between the two lines (DE-lncRNAs-2L) at different time points.

The transcriptome assembly yielded 339,659 transcripts and 262,716 gene loci in total, which revealed 73,992 novel transcripts and 9446 novel genes not annotated from the reference genome. A total of 91, 202, 323, and 196 differentially expressed lncRNAs (DE-lncRNAs) were identified between the four different time points vs. 0 hai, respectively, in line 540. A total of 45, 142, 304, and 166 DE-lncRNAs were found for the respective time points in line 664. The lncRNA profile changes followed a similar pattern to that of mRNA, showing a peak at 12 hai. In both lines, the number of induced lncRNAs was more than repressed ones at 3 hai, while there were more repressed lncRNAs than induced ones at later time points (Figure 5A). Additionally, a total of 120, 271, 490, and 306 lncRNAs were differentially expressed between the two lines (DE-lncRNAs-2L, Figure 5B, Table S4). From the numbers above, it is clear that both lines respond to the orange rust infection most actively at 12 hai at the lncRNA level.

### 2.4. sRNAseq Analysis

The sequencing of small RNA (sRNA) libraries from the two lines, 540 and 664, at five different time points (0, 3, 6, 12, and 24 hai) with three biological replicates (30 samples in total) generated a total of 826,391,458 raw reads, with an average of 27,546,381.93 reads per sample. The read lengths ranging from 18 to 35 nt were kept, and a peak of 24 nt was observed in most of the samples (Figure S3). The clean reads were further classified as rRNA, tRNA, mature miRNA, pre-miRNA, other RNA, and unknowns (Table S5). In most of the samples, rRNA was the largest portion, with an average proportion of 45.34% in the small RNAseq data. No novel miRNA was identified.

The differentially expressed sRNA analysis was conducted by comparing the sRNA expression level between samples at 3, 6, 12, and 24 hai, respectively, and the sample at 0 hai for each line. A total of 29 differentially expressed miRNAs (DEMs) within 21 miRNA families were identified. All of these 29 DEMs were identified as differentially expressed between the two lines as well (DEM2L, Table S6). Each DEM2L had targets on more than several thousand potential genes.

### 2.5. Weighted Correlation Network Analysis (WGCNA)

**Figure 6:**
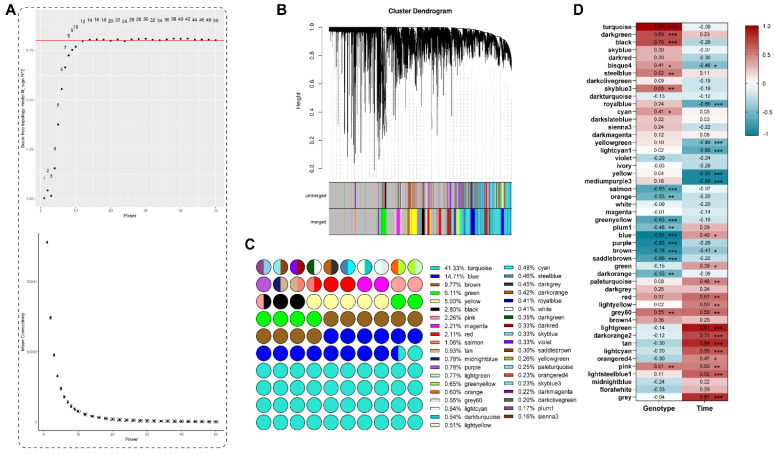
(**A**) Selection of soft-thresholding powers (β). (**B**) Gene co-expression network gene clustering numbers and modular cutting. (**C**) The proportion of hub genes from different modules (grey module was excluded). (**D**) Association analysis of gene co-expression network modules with genotype/resistance ability and time-dynamic traits. There are two numbers in each unit: the upper one is the correlation value and the lower number is the *p*-value. (* *p* < 0.05, ** *p* < 0.01, *** *p* < 0.001).

All mature miRNAs, mRNAs, and lncRNAs were combined for the WGCNA. A weight value, β = 14 (Figure 6A), was selected for adjacency matrix construction. A total of 49 co-expression modules were obtained (Figure 6B–D), out of which 2 (turquoise and blue) were significantly related to genotype/resistance ability and 1 (light green) to inoculation time points (Figure 6D).

The turquoise module had, by far, the greatest number of genes (15,792, with 4374 as hub genes) compared to any other module, including 14,860 mRNAs (4260 hubs), 834 lncRNAs (110 hubs), and 98 miRNAs (4 hubs) (Table S7). The blue module had 5324 total genes (1137 hubs). Of those, 4967 were mRNAs (1110 hubs), 300 were lncRNAs (27 hubs), and 57 were miRNAs (0 hubs) (Table S7). GO annotations of the hub genes in two modules, turquoise and blue, revealed that most of them were related to “response to stimulus”, “immune system process”, and “detoxification”, which confirmed the relationship between these two modules and SORD response.

**Figure 7:**
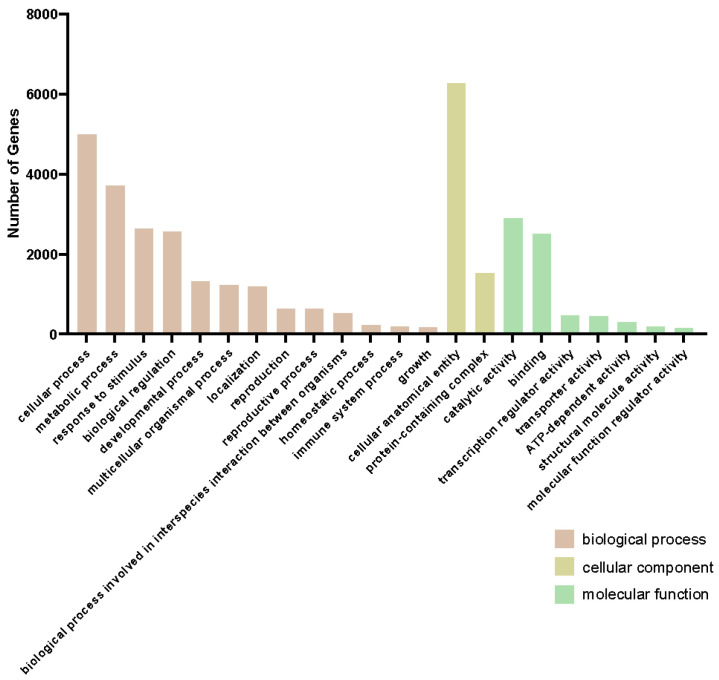
GO enrichment of turquoise module. Top significantly enriched GO terms in the biological process category. Cellular components and molecular functions are shown for genes in the turquoise module. Bar plots represent the number of genes of different GO terms in the turquoise module.

**Figure 8:**
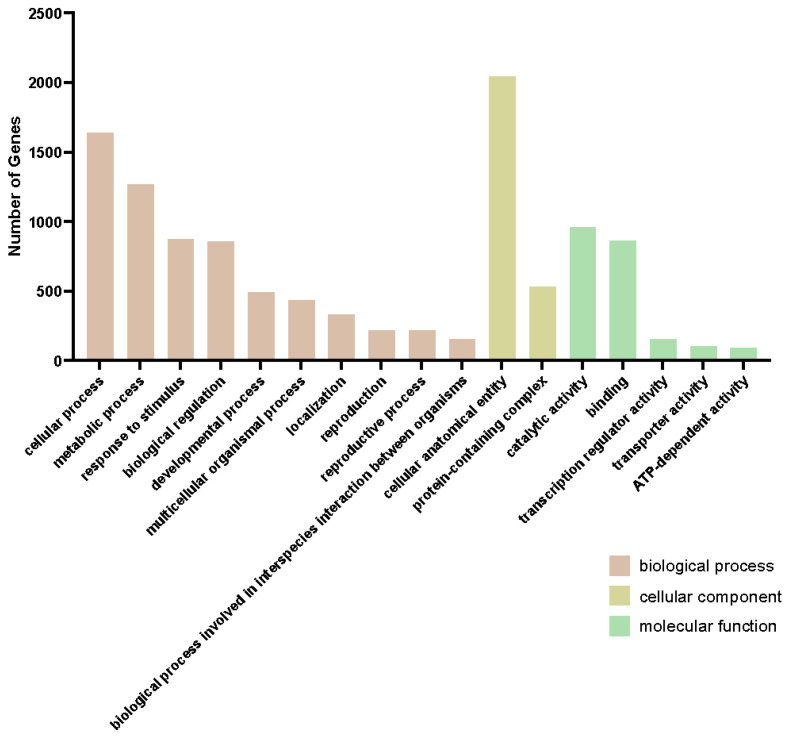
GO enrichment of blue module. Top significantly enriched GO terms in the biological process category. Cellular components and molecular functions are shown for genes in the blue module. Bar plots represent the number of genes of different GO terms in the blue module.

**Figure 9:**
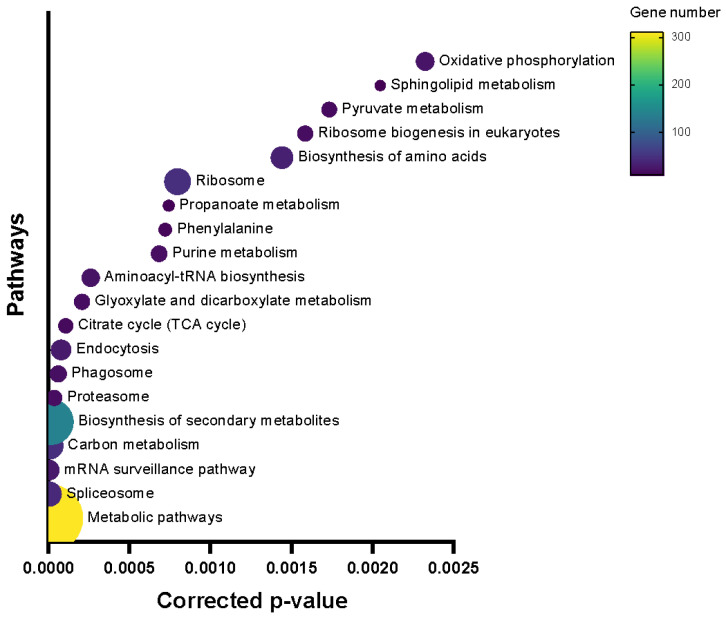
Top 20 metabolic pathways with the smallest *p*-values of the blue module identified by KEGG enrichment.

The genes in the blue module were co-expressed at a higher level in the resistant line 540 than the susceptible line 664, while the genes in the turquoise module were co-expressed at a higher level in line 664 than 540. Overall, the GO term landscape was very similar between the turquoise and blue modules (Figure 7 and Figure 8). This suggests that these modules involve similar biological and molecular processes, but these behave differently between the two lines. In molecular function GO terms, the turquoise module has three unique GO terms: “nutrient reservoir activity”, “protein-containing complex destabilizing activity”, and “small molecule sensor activity”. To better understand which metabolic processes occur in the blue module, we performed KEGG pathway enrichment (Figure 9).

The top three enriched pathways in the blue module include “glycosphingolipid biosynthesis—globo and isoglobo series”, “other types of O-glycan biosynthesis”, and “betalain biosynthesis”. Also, a few biologically interesting pathways were identified: “tropane, piperidine, and pyridine alkaloid biosynthesis” and “SNARE interactions in vesicular transport”.

The top three enriched pathways in the turquoise module included “porphyrin metabolism, “propanoate metabolism”, and “photosynthesis—antenna proteins”. We also identified the “autophagy” pathway as being biologically interesting for the SORD response. The “porphyrin metabolism” and “photosynthesis—antenna proteins” pathways are interesting since they relate to the synthesis of chlorophyll (derived from porphyrin backbone) and antenna proteins, which are critical for photosynthesis. Meanwhile, the “propanoate metabolism” and “autophagy” pathways are interesting since they relate to amino acid oxidation and nutrient recycling in the cell.

**Figure 10:**
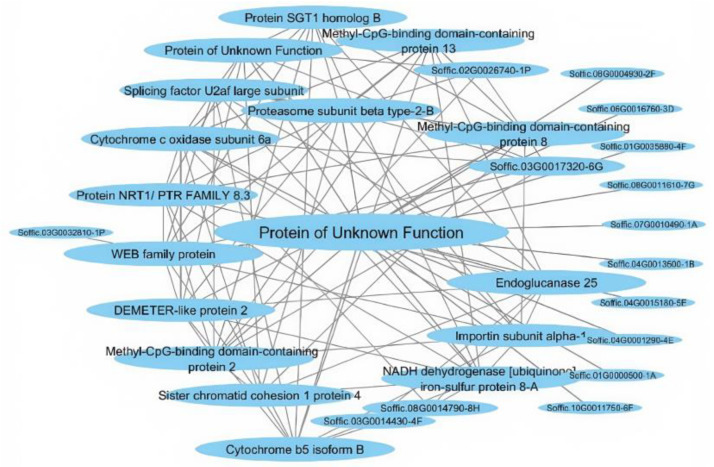
Blue module network. The selection was made by first identifying the top 10 genes with the strongest interactions, followed by selecting up to 10 of the strongest interactions for each of these genes, resulting in a maximum of 100 interactions in total.

To better understand the architecture of the most significant interactions in the blue module, the most significant sub-networks were extracted. *Soffic.02G0011070-3C,* encoding a protein of unknown function, had the most interactions in the sub-network (Figure 10).

The top three unique mRNA–lncRNA interactions in the blue module were lncRNA *MSTRG.50654* with mRNA *Soffic.02G0011070-3C*, encoded for a protein of unknown function; lncRNA *MSTRG.49194* with mRNA *Soffic.01G0009980-4E*, which encoded for Protein SGT1 homolog B; and lncRNA *MSTRG.107691* with mRNA *Soffic.01G0020660-2B*, which encoded for Pre-mRNA processing factor 19 homolog 2.

A total of 202 mRNAs and 2 miRNAs as hub genes were also DEG2L or DEM2L (Figure S4A,B). A total of 10 genes (Soffic.02G0012580-8H, Soffic.02G0012750-6H, LAp.02H0013130, LAp.02H0013490, Soffic.02G0005030-6G, Soffic.02G0013620-3F, LAp.02F0013720, Soffic.02G0013820-5F, Soffic.02G0014000-6F, and Soffic.02G0014380-5F) around the Chr2-G1 marker were defined as DEG2L or hub genes.

### 2.6. Detection of Genomic Variation

A total of 908,844 SNPs located on 67,465 genes were identified between the two lines. About 5474 hub genes, 3670 DEG2Ls, 3011 hub genes in the turquoise module, and 742 hub genes in the blue module contain SNPs (Figure S4A,C). Among the expressed genes, the numbers of SNPs on Chr01, Chr02, Chr03, and Chr04 were larger than those on the rest of the chromosomes, which was consistent with the DEG2L distribution (Table S3). About 93 genes with SNPs were located within a sequence window from 200 kb upstream to 200 kb downstream of the G1 marker, which provided a great marker source for further fine-mapping the candidate genes linked by the G1 marker.

### 2.7. Metabolomic Analysis

To explore the metabolite variations between the two lines, 540 and 664, in response to SORD, metabolites were extracted from the two lines without infection and after orange rust infection. The first batch of seedlings did not show symptoms until three weeks later; thus, the samples subjected to LC/MS global metabolomic analysis were collected from the second batch of seedlings three weeks after the SORD infection. The global metabolomic analysis resulted in a total of 2729 characteristics or metabolites identified and quantified, including 971 characteristics from the positive mode and 1758 from the negative mode.

Principal component analysis (PCA) and partial least squares–discriminant analysis (PLS-DA) demonstrated the high repeatability of biological replicates and high variability between the mock and inoculated groups in the two lines (Figures S5 and S6). The heatmap of the top 50 identified metabolites was clustered and showed changes between the two lines (Figure S7).

**Figure 11:**
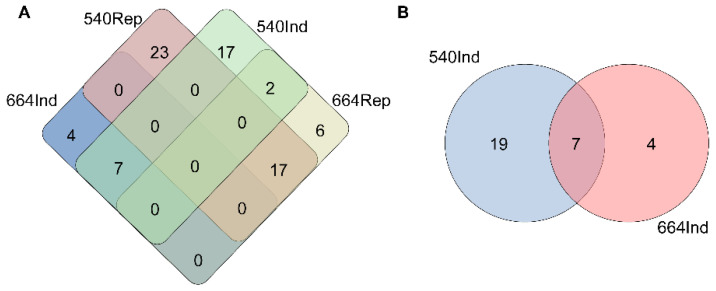
(**A**) Venn diagram of the induced significant and repressed significant metabolites in two sister lines (resistant line 540 and susceptible line 664) after inoculation. (**B**) Venn diagram of the induced significant metabolites in two sister lines (line 540 and line 664).

The Venn diagram based on the differentially produced metabolites (Table S8) of the two lines after the inoculation revealed 7 up-regulated and 17 down-regulated metabolites detected jointly in both lines, and another 2 metabolites were induced in the resistant line (540) but repressed in the susceptible line (664) (Figure 11A). Additionally, in the resistant line 540, 19 unique induced metabolites (Figure 11B, Table 1) had the potential to contribute to orange rust resistance.

### 2.8. Transcriptional and Metabolomic Joint Analysis

**Figure 12:**
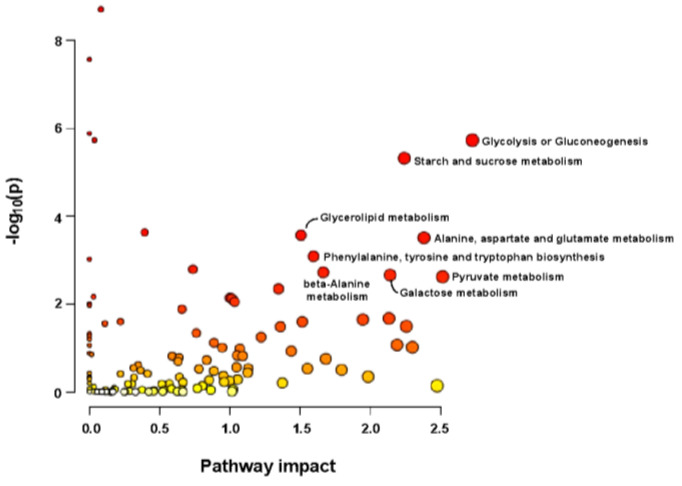
Joint analysis of transcriptome and metabolome illustrating the overview of pathway analysis based on DEGs and induced metabolites in line 540 according to the *p*-values from the pathway enrichment analysis and pathway impact values from the pathway topology analysis. (A stronger red color indicates higher statistical significance).

**Figure 13:**
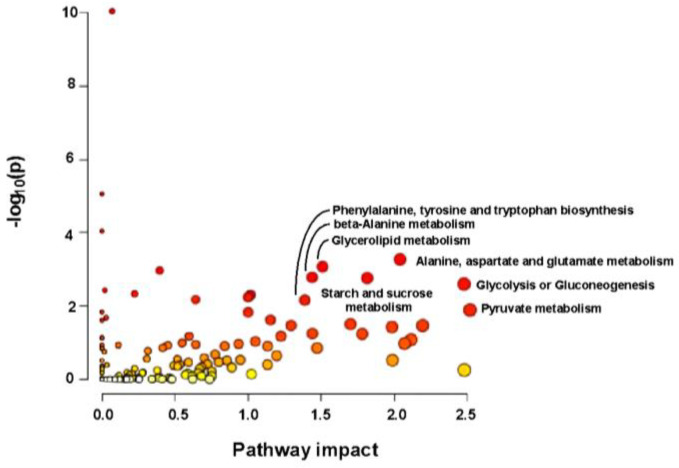
Joint analysis of transcriptome and metabolome illustrating the overview of pathway analysis based on DEG2Ls and induced metabolites in line 540 according to the *p*-values from the pathway enrichment analysis and pathway impact values from the pathway topology analysis. (A stronger red color indicates higher statistical significance).

A joint analysis of DEG expression at all time points in line 540, as well as the DEG2Ls and significantly induced metabolites in the resistant line (540) revealed significant enrichment in various pathways (Figure 12 and Figure 13). The results illustrated that in the resistant line, the rate of sugar metabolism is significantly faster, leading to more sugar accumulation. Plus, the “phenylalanine, tyrosine, and tryptophan biosynthesis” pathway was among the most significant pathways, which is of great interest as previous studies have reported that phenylalanine prevents fungal infection on fruits in the post-harvest stage [34].

## 3. Discussion

*Puccinia kuehnii*, causing sugarcane orange rust disease, is one of the major fungi threatening sugarcane production this century. To protect sugarcane production from orange rust and reduce yield losses, we aimed to identify resistance genes for breeding programs. Though we previously identified a major QTL controlling SORD resistance [24], this is far from candidate gene identification given the complex genome of sugarcane. To pave the road for a fine-mapping effort, in this study, we selected two sister lines with contrasting response to SORD and differing in genotype in the major QTL qORR109 [24] for comparison at the morphological, transcriptomic, and metabolomic levels to overview the possible factors contributing to the SORD resistance underlying the major QTL. The findings provide a comprehensive framework, insights, and a foundation to uncover the unknown stories behind OR resistance in subsequent research.

### 3.1. Morphological Analysis

The results of this study showed significant morphological differences between the two lines with contrasting resistance levels to SORD, including the lower stomata density and smaller stomata size on the leaf surface of the resistant line compared to the susceptible line. These results suggest that morphological variance cannot be excluded as a contributor to the different resistance responses of the two lines.

The pathogen can invade plants through natural openings, such as the stomata, lenticels, and hydathodes. Most plant pathogens can bypass the epidermal barrier and utilize the stomata as entry points to invade plant tissues, since among the natural openings, stomata dominate in the aerial parts of plants. As a defense mechanism, smaller stomatal apertures and less stomata density can eliminate or restrict pathogen invasion. Previous studies indicated that initial stomatal closure is triggered by plants sensing microbe-associated molecular patterns (MAMPs) and damage-associated molecular patterns (DAMPs) [35], as evidenced by the presence of DAMP-related genes in the DEG list and the turquoise module in this study. Stomatal closure/dynamics after infection can be further investigated to understand the stomatal involvement in disease defense.

### 3.2. Transcriptomic Analysis

By performing transcriptomic analysis based on mRNAs, miRNAs, and lncRNAs, for the first time, we identified protein coding and regulatory genes responding to orange rust within 24 hai, which provided a decent database to understand the plant response to orange rust disease at the early infection stage. Moreover, comparing the DEGs between the two lines with contrasting responses to SORD (DEG2L) defined eight sub-cases (Table S2), which provided a more comprehensive gene pool to understand how the resistant line responded to early orange rust disease infection differently from the susceptible line. The abundance of DEG2Ls observed at 12 hai for both lines suggested that the plants had peak molecular and physiological responses against SORD at 12 h after infection.

Most DEG2Ls had higher expression in the resistant line 540 than in 664. The plant immune response involves the fine-tuning of a plethora of cellular processes to optimize plant defenses. These include optimizing basic cell processes, such as protein translation, photosynthesis, energy metabolism, specialized metabolite metabolism, osmotic homeostasis, ROS signaling, intracellular signaling, and intercellular signaling, and specific defense response pathways. Below, we discuss the expression patterns and potential functions of some specific DEG2Ls during early response to SORD.

Two DEG2Ls encoding phosphomethylpyrimidine synthase and thiamine thiazole synthase 2 were both significantly induced in line 540 at 6 hai and 12 hai and were both involved in the biosynthesis of thiamine (Vitamin B1), suggesting that the synthesis of this vitamin is important in SORD response. In general, thiamine is a necessary cofactor for many enzymatic reactions. Il-Pyung et al. (2007) showed that the application of thiamine to *Arabidopsis*, followed by infection with *Pseudomonas syringae,* exhibited almost no symptoms of infection, which proved that thiamine “primed” the plant’s defense responses, thereby reducing the metabolic cost of the response [36]. Furthermore, this thiamine-induced immune priming is dependent on cell ROS content and NPR1 induction, and NPR1 content was higher in line 540 as a DEG2L. So, it is plausible that line 540 has increased thiamine biosynthesis, which contributes to its resistance by increasing metabolic efficiency. However, it is possible that thiamine is biologically active at low concentrations, as it was not detected by the metabolomic assays.

Agmatine coumaryl transferase was a DEG2L annotated with the “catalytic activity” GO term, and is a critical enzyme in the biosynthesis of hordatine A and B, which is of particular interest due to its antifungal properties, and has been reported to have a drastic inhibitory effect on fungal spore germination [37]. Its 3 hai peak was drastically higher in line 540, which suggests that hordatines may be part of the chemical arsenal against SORD. We suspect these genes play a strong role in the direct response to SORD. However, in addition to direct chemical defenses, there is a lot of evidence in the DEG2L pool that line 540’s resistance stems from optimizing basic housekeeping and signaling processes. The optimization of housekeeping functions in line 540 is represented by DEG2Ls such as small ribosomal subunit protein S4-3, aquaporin TIP4-1, and calcium-transporting ATPase 11. This suggests that the modulation of protein translation, osmotic pressure, and membrane cation transport plays a fundamental role in priming cells’ response to SORD [38,39,40]. Cell signaling is also critical for optimal stress response. For example, the DEG2Ls copper-containing amine oxidase gamma 2 and thioredoxin-like 3-1 are crucial genes responsible for nitric oxide signaling and protein glutathionylation, respectively [41,42,43]. All the aforementioned DEG2Ls were expressed at significantly higher levels in line 540.

Overall, the DEG2Ls showed significant enrichment in “Monoterpenoid biosynthesis” and “Limonene and pinene degradation”. Interestingly, limonene and pinene are common monoterpenoids and are classified as volatile organic compounds (VOCs). These findings suggest these monoterpenoids are being synthesized—and likely emitted—and at the same time, they are being detected, uptaken, and degraded. Much research is underway showing terpenoids as a means of chemical communication between plants, both through air and roots [44,45]. The emission of these VOCs may be a means of alerting neighboring plants of an active infection.

Besides mRNA, non-coding RNA (including miRNA and lncRNA) can act as both a positive and negative regulator in plant immunity temporally and spatially, thereby modulating the immune responses of plants [46]. In this study, 29 DEM2Ls within 21 miRNA families were identified, including miR396, miR408, and miR528, which were reported to have the functions of regulating the defense against fungi and bacteria [47,48,49]. Long non-coding RNAs may have as much regulatory power as miRNAs and the capacity to alter the transcriptomic landscape involved in biotic and abiotic stress responses [50,51]. To the best of our knowledge, these lncRNAs are the first attempt at a whole-transcriptome annotation of the lncRNAs in sugarcane. We observed that line 540 had a much higher transcriptomic response in terms of lncRNAs compared to line 664. The WGCNA showed a plethora of lncRNAs as hub genes with direct correlations to mRNA. In the blue module, which had genes co-expressed at much higher levels in line 540, the number of hub-lncRNAs outweighed the number of hub miRNAs. These results strongly suggest that lncRNA regulation is a part of the SORD resistance response.

The blue module represents a group of genes that are more co-expressed in the resistant line 540 than in the line 664. In the blue module, two of the top three enriched KEGG pathways were related to the cell wall or cell membrane modifications, “O-glycan biosynthesis” and “glycosphingolipid biosynthesis”. The cell wall is the first interface between the fungal pathogen and the host. It represents the first defense barrier against infection. Cell wall and membrane modifications often occur through modifying the glycome of the associated structure or associated proteins and lipids (i.e., glycoproteins, glycolipids). In most organisms, these carbohydrate modifications are presumed to be essential for host–pathogen recognition and cell–cell signaling. Although their biosynthesis in plants is well described, their exact biological roles in plants are arguably the least known because mutations are typically lethal. Glycosphingolipid biosynthesis, an enriched blue module pathway, is thought to be responsible for proper protein packaging in the Golgi apparatus, and abnormal sphingolipid mannosylation induces salicylic acid-mediated cell death in *Arabidopsis* [52,53]. However, the enriched Hexosaminidase 1 in this pathway is responsible for the N-glycosylation of cell wall proteins and is required for paucimannosylation [54]. N-glycosylation is often implicated in protein localization and folding into the cell wall and membrane. Documented mutations in enzymes that synthesize these N-glycans severely affect plant development, growth, and embryo viability [55,56]. However, Häweker et al. (2010) demonstrated that the reduced N-glycosylation of PAMP receptors actually severely decreased receptor abundance and ligand recognition at the cell surface, and these receptors are critical to elicit adequate oxidative bursts against the pathogen [57]. We presume that line 540 has an increased ability to perceive fungal PAMPs such as chitin and chitosan, and may have an increased abundance of PAMP receptors at the cell surface due to this increased N-glycosylation. Aside from cell wall modifications, *Soffic.01G0009980-4E*, which coded for Protein SGT1 homolog B, was predicted to interact with lncRNA *MSTRG.49194*. Protein SGT1 has been documented to be involved in plant innate immunity by compounding resistance with R (resistance) proteins, and aids in the recognition of fungal pathogens like downy mildew in *Arabidopsis* [58,59]. However, Fan et al. (2020) discovered that the SGT1 protein in rice is regulated by non-coding circular RNAs, which induce its expression [60]. Although it is difficult to say whether *MSTRG.49194* is a circular RNA, it is very plausible that it induces SGT1 expression and compounds the defense response to SORD in line 540.

By further investigating the blue module, we found out that ROS also played an important role in the defense against SORD. Extradiol ring-cleavage dioxygenase was in the enriched “betalain biosynthesis” pathway and is a critical enzyme for the synthesis of other molecules known as arabidopyrones from caffeate [61], which are molecules gaining popularity due to their possible antioxidant role in plant defense. Additionally, the “tropane, piperidine, and pyridine alkaloid biosynthesis” pathway in the blue module suggests that line 540 may employ alkaloids as ROS scavengers during the oxidative burst to protect its proteins and nucleic acids. Alkaloids have a wide variety of functions in plants, but their role as a non-enzymatic antioxidant is quite prominent [62], and this ROS-scavenging ability is also believed to confer antifungal properties, even at low concentrations [63,64]. Many alkaloids are synthesized from phenylalanine, an aromatic amino acid, which can also be modified to produce polyphenol constituents as strong antioxidants [65]. We found several phenolic compounds with purported antioxidant capabilities in our metabolomic assays, which we will discuss later. However, we believe these phenylalanine derivatives to form a large component of the direct defense against SORD in line 540. A large volume of the scientific literature supports that the antioxidant capacity of the plant host correlates with its ability to resist pathogens. On the other hand, genes encoding Cationic peroxidase SPC4 (*Soffic.03G0039230-1A*) and Glutathione S-transferase (*Soffic.09G0001590-2B*) were two interesting DEG2Ls with enzymatic ROS-scavenging abilities, and were both highly induced in line 540. These results indicated that enzymatic antioxidant mechanisms may also contribute to line 540’s antifungal properties [66,67].

### 3.3. Metabolomic Analysis

In this study, we conducted a metabolomic analysis on mock-inoculated samples vs. pathogen-inoculated samples on both lines and identified 19 unique induced metabolites in the resistant line (line 540). To eliminate environmental noise and target the key pathways, information on these changed metabolites, along with DEG2Ls and hub genes, was used to conduct a joint analysis to highlight resistance-associated metabolic pathways. The “phenylalanine, tyrosine, and tryptophan biosynthesis” pathway, the phenylpropanoid pathway (the pathway that processes phenylalanine derivatives), and sugar-related pathways were notable. It has been reported that the amino acid phenylalanine could induce a plant defense response against *Botrytis cinerea* in petunia and tomato leaves, and phenylalanine has also been demonstrated to mitigate fungal diseases in the post-harvest phase [68]. Phenylalanine could be used as an environmentally friendly fungal protectant since no phenylalanine residue was detected on/in the fruit after spraying it [34]. However, the phenylpropanoid pathway is extremely vast and converts phenylalanine into a plethora of large aromatic molecules, such as flavonoids and polyphenols [30]. It is also heavily involved in plant immune hormone biosynthesis, such as salicylic acid. These phenylpropanoid pathway derivatives have strong antioxidant capacity, which supports the assumption that the phenylpropanoid pathway may be an active resistance factor against SORD. For example, wheat rust (*Puccinia triticina*) spore germination is inhibited by neutralizing reactive oxygen species produced by the pathogen using antioxidants [69]. It would also explain why line 540 has more phenylpropanoid derivates than line 664, such as ferulate, 3-hydroxyphenylacetate, and 4-hydroxyphenylacetate (Table 1). Interestingly, caffeate was also induced, but in both lines. Caffeate is a precursor of ferulate [70], which suggests that the conversion of caffeate into ferulate may be important for SORD resistance. The genes of the pathway may not necessarily all be co-expressed equally. Some genes in the same pathway may even have opposite expression profiles depending on cellular demands. This may explain why this pathway was not annotated in a module from WGCNA. The enrichment of the phenylpropanoid pathway and its metabolic derivatives suggest that SORD resistance may be (at least partly) explained by the production of aromatic phenylpropanoid derivatives. Preliminarily, phenylpropanoid derivative biosynthesis would agree with the resistance mechanisms discovered in resistant varieties of other species. For example, wheat resistance to *Alternaria* blight was explained by a significantly high polyphenol content [71].

Significant changes were also seen in sugar metabolites (Table S8). The role of carbohydrate metabolism in plant disease resistance is not as clear and likely involves multiple cellular functions and pathways. Sugars are known to regulate salicylic acid and jasmonic acid accumulation, but also regulate plasmodesmatal development, PAMP priming, and programmed cell death [72]. However, one interesting observation is that raffinose is induced in line 540. Raffinose is a very soluble carbohydrate used in carbon transportation and delivery across the phloem and is associated with improved stress tolerance [73]. From our morphological observations, line 540 had a higher phloem density, which hints at a higher transport capacity of nutrients. Increased carbon transport across the phloem may help fuel higher metabolic demands in the leaves of line 540 for polyphenol biosynthesis. Additionally, SORD resistance may simply confer higher energy demands that warrant increased carbon transportation across the phloem. It is important to note that metabolites such as ferulate and raffinose may be upstream metabolites of the molecule that confers resistance. Active metabolites may require a much lower concentration for their activity, or may have too high molecular weight, and thus may be undetectable using standard metabolomic assays. Nevertheless, the genes that regulate these metabolites were observed to be changed in the transcriptomic data. Thus, the metabolic results in this study are generally consistent with the transcriptomic results.

### 3.4. Limitations and Future Directions

Although there are important discoveries revealed by these studies, there are some limitations: (I) Based on the significantly contrasting resistant ability of 173 F1 progeny, derived from a cross between sugarcane clones CP95-1039 and CP88-1762, the two lines used in this project (line 540 and line 664) were identified, because these two lines are only different in the major QTL qORR109 in terms of genotype [24]. While this situation enables a focus on the thorough study of the major QTL, it ignores the additive and epistatic effects with and among other minor QTLs, or other regions conferring SORD resistance. (II) Though significant differences at morphological, transcriptomic, and metabolic levels were found between the two lines, our results lack data on the original parental lines, CP95-1039 and CP88-1762, and other individuals in the progeny. Thus, no genetic analysis could be conducted to pinpoint the specific variation underlying the contrasting phenotype, which will be conducted in a future experiment. (III) The number of DEGs/DEG2Ls was the highest at 12 hai. However, these DEGs/DEG2Ls were possibly due to responses to circadian rhythm and the differences in other regions in their genomic background. Eliminating the impact of circadian rhythm to unveil essential genes remains a critical research challenge. (IV) Non-novel miRNAs were probably predicted for two reasons: (1) Rice and sorghum small RNA databases were chosen, which have been fully annotated, as rice has been widely studied as a monocotyledonous model species. (2) Putative sugarcane miRNA was annotated according to its alignment to rice + sorghum miRNA with two mismatches, which could limit the power to predict novel miRNA sequences since the novel miRNA sequences already exist in the “two mismatch” sequences. (V) The specific lncRNA–mRNA interactions could not be explained since lncRNAs have diverse mechanisms of regulation that are often sequence-independent and more dependent on their secondary structures [51]. However, the analysis did provide a hierarchical list of annotated lncRNAs that have a strong association with the SORD resistance phenotype, which can be studied further for their function in other follow-up studies. (VI) Numerous sugar-related metabolites or pathways in the transcriptome and metabolome are assumed to be significantly associated with resistance. However, it is challenging to identify which metabolite or pathway is considered to be the key sugar metabolite because these pathways interact with each other and with other cellular processes. (VII) At this stage, we could merely propose the potential possibility of these compounds without identifying them from plants or from the pathogen. A subsequent phase of our research will need to be conducted to prove whether these compounds show inhibitory effects on *P. kuehnii*. (VIII) Even though plenty of DEGs, DEG2Ls, and hub genes were identified, the real key gene around the G1 marker may not respond in the first 24 hai. The RNAseq data only tell a story at the transcript level [19]. Thus, fine mapping is still needed in further studies.

Given the above limitations, further research is needed to elucidate the morphological differences between the two lines and their role in early-stage fungal invasion, as this remains an underexplored aspect of host–pathogen interactions. Our RNA-seq analysis provided a reference resource of genes responding to the early infection of SORD, as plant disease resistance response can be a dynamic process. To identify reliable candidate genes linked to SORD resistance in the major QTL qORR109, further genetic mapping, fine mapping, cloning, and candidate gene characterization are still needed to elucidate the mechanism of the key gene’s functions. The metabolite analysis found 19 unique induced metabolites in the resistant line (540), providing valuable insights into the varied metabolite pathways associated with SORD resistance. Combining metabolomic and transcriptomic analyses showed that the sugar and phenylalanine biosynthesis metabolism pathways were highlighted in the SORD-resistant line. Therefore, another important experiment in the future is to validate the potential antifungal functions of these metabolites for developing a sustainable disease control approach. In conclusion, the results of this study provide a large amount of genetics and multi-omics datasets enriched with potential candidates of SORD resistance and pave the road for downstream fine-mapping efforts to identify the candidate genes controlling SORD resistance and their functional characterizations.

## 4. Materials and Methods

### 4.1. Plant Materials

Previously, a population of F1 hybrid clones was derived from a cross between the sugarcane clone CP95-1039 and the sugarcane cultivar CP88-1762, which showed resistance and susceptibility to SORD, respectively. This population was developed by the Sugarcane Field Station, United States Department of Agriculture (USDA), Agricultural Research Service (ARS) at Canal Point, FL, USA. This F1 population was used for SORD resistance QTL analysis previously [24], and three major QTLs were identified. Among these F1 populations, two individual lines were identified, 540 and 664, having resistant and susceptible genotypes and phenotypes of the QTL qORR109 to SORD, respectively, but having susceptible genotypes at the other two QTL regions. Thus, these two sister lines were presumably fixed at the other two QTL regions, but were only different at the major QTL qORR109 region, which contributed to the contrasting phenotype of resistance. These two lines were planted at Canal Point in 2019 and at the University of Florida in 2022, in the greenhouse for experiments in this study. The observation of cross-sections of the leaves was based on cryosections.

### 4.2. Sugarcane Inoculation

Sugarcane seedlings of the two lines were established in a greenhouse with a temperature of 30 °C, natural light, and a soil moisture level of 70%. Three replicates of each line for each sampling time and control were planted in seedling trays. Urediniospores were vacuum-collected from the abaxial surface of symptomatic leaves [74]. To prepare the inoculum, urediniospores were suspended in sterilized deionized water containing 0.002% l nonanol and 0.01% Tween 20 solution, and the concentration was adjusted to 10^5^ urediniospores/mL [74]. The concentration of the inoculum and the germination rate of the urediniospores was assessed with a hemocytometer. The inoculum was used soon after it was prepared. About 200 µL of inoculum was placed, starting at 7 a.m.

Two-month-old seedlings were inoculated by placing 200 μL of inoculum into the leaf whorl using a pipette at 7 a.m. on the day of sampling [74]. The control seedlings were mock inoculated similarly, but with an inoculum solution free of urediniospores.

### 4.3. Morphological Observation

The newest non-inoculated unfolded leaves (3rd or 4th) were collected from at least three seedlings as biological replicates to assess leaf stomatal density and size. For each leaf, both the adaxial and abaxial surfaces at the leaf base were observed. For stomata observation, the leaf surface was printed on dried nail polish and observed under a Leica CTR 4000 microscope (Leica Microsystems, Wetzlar, Germany). Six areas of each nail polish print were randomly selected, and the number of stomata was observed at a 200× magnification and averaged in the six areas for each sampled leaf surface. The stomata density per mm^2^ was calculated for each area and averaged for each sampled leaf surface. The length and width of the stomata were measured under the microscope at 400× magnification. Stomata si/zes are represented by length × width. The size of at least five stomata from each sampled leaf surface was measured and averaged for data analysis. Leaf width (the widest part) was also measured at the same time. The statistics analysis was carried out using a Student’s *t*-test.

### 4.4. RNAseq Library Construction and Sequencing

At 0, 3, 6, 12, and 24 hai, the base of the newest 1st and 2nd leaf samples with inoculated spores attached in the leaf whorl were dissected quickly, collected, and stored in liquid nitrogen. At each time point, leaf samples from three different seedlings were collected as three biological replicates. The total RNA was extracted from the samples using the Direct-zol kit from Zymo Research (Zymo Research, Irvine, CA, USA). RNA quality and quantity were checked using a Bioanalyzer (Agilant Corporation, Inc., Santa Clara, CA, USA). About 6 μg of total RNA sample of good quality from each sample was submitted for library construction and sequencing at Novogene (Novogene Corporation Inc., Sacramento, CA, USA). Libraries for both sRNA and mRNA sequencing were constructed using the Small RNA Sample Pre Kit (Illumina, San Diego, CA, USA), and whole-transcriptome sequencing was undertaken using an Illumina high-throughput sequencing platform (PE150, Q30 ≥ 80% for mRNA libraries and SE50, Q30 ≥ 85% for sRNA).

### 4.5. mRNAseq Analysis

The clean reads with a length above 75 bp after Trimmomatic v.0.39 [75] trimming at default settings were kept. SortMeRNA v.2.1 with default settings [76] was used to remove the contaminated rRNAs. Clean reads of each sample were aligned to the *S. officinarum* genome (https://ftp.ncbi.nlm.nih.gov/genomes/all/GCA/020/631/735/GCA_020631735.1_ASM2063173v1) (accessed on 25 November 2022) using Hisat2 v.2.2.1-3n following the default settings [77]. SAM files generated from His/at2 were converted to BAM files through samtools-view [78], which were input into FeatureCount (v. 2.0.3) [79] to count the gene read number. The gene reads of all libraries were organized and input into the DEseq2 program [80] in the R package to identify the differentially expressed genes (DEGs if the |log2(fold change)| > 2 where fold change = experimental value/control value, padj < 0.05) between samples at 3, 6, 12, and 24 hai vs. 0 hai, respectively, in each line. Genes presented in 25% of samples and with more than 5 counts were considered as expressed genes and included in the DEG analysis of lines 540 and 664 separately. The DEGs identified in lines 540 and 664 were further compared to identify the DEGs between these two lines, denoted as DEG2Ls, by comparing the fold change value generated from the DEseq2 of each gene at each time point between the two lines. A DEG2L was defined if any one of the two broad scenarios below happened (the two broad scenarios can be further divided into eight sub-cases):

- If a gene was a DEG in one line at a time point but not expressed or not a DEG in the other line, then this gene was defined as a DEG2L. (This DEG2L in this scenario suggests that a particular DEG undergoes a significant change in expression in only one of the lines.)
- If a gene was expressed in both lines and was a DEG of either line at a time point, but the FC values (the fold change of gene expression between the given time point and 0 hai) between the two lines were different by more than three times, or the DEG had different expression trends between two lines, then this gene was defined as a DEG2L. (The DEG2L in this scenario suggests that a certain DEG exhibits a greater magnitude of change in one line, or exhibits opposing trends of change between the two lines.)

DEGs and DEG2Ls were further annotated by blasting with the rice database (MSU v.7). GO and KEGG enrichment were conducted based on the GO annotation and KEGG annotation in the gene function table. KEGG enrichment visualization is achieved through KOBAS [81]. Moreover, since *S. spontaneum* still accounts for 10–20% of the genome of modern sugarcane cultivars, sequence reads, which were not aligned to the *S. officinarum* reference genome, were further aligned to the *S. spontaneum* genome [33] using Hisat2 v.2.2.1-3n [77] with default settings, so as not to lose any genes.

### 4.6. LncRNA Data Analysis

LncRNA transcripts were predicted and annotated using the FEELnc software (v.0.2.1) package [82]. The BAM files generated from Hisat2 were assembled into a new whole-transcriptome annotation using the Stringtie2 assembler software (v.1.3.6) with default settings [83]. This new transcriptome contained previously annotated mRNAs, as well as novel transcripts, which are potentially lncRNAs. This new whole-transcriptome annotation served as an input for FEELnc. FEELnc can be divided into 2 modules: FEELnc.filter and FEELnc.codpot. FEELnc.filter was run with default parameters, which removed any transcripts overlapping with a sense exon with an annotated mRNA. The FEELnc.codpot function provided a training set of 200 mRNAs and 200 non-coding RNAs from *Sorghum bicolor* to extract k-mer frequencies. The first 200 lncRNAs and mRNAs were downloaded from the Sorghum gene database (NCBIV3) of the NCBI (https://www.ncbi.nlm.nih.gov/datasets/gene/taxon/4558/?gene_type=non-coding) (accessed on 25 November 2022). The output of FEELnc is an annotation file with only predicted lncRNAs, to which read counts can be mapped for further analysis. Differentially expressed lncRNAs and differentially expressed lncRNAs between two lines were defined in the same way as the mRNA and miRNA datasets.

### 4.7. sRNAseq Data Analysis

Similar to mRNA, Trimmomatic v.0.39 [75] was used with default settings for single-end sequencing to remove poor-quality reads. Clean reads of 18–35 nt were retained and further classified by searching in the RNAcentral database (accessed on 26 January 2023, https://rnacentral.org/about-us) with different types of RNA molecules, particularly from) rice and sorghum small RNAs with two mismatches. The unaligned reads were put into miRDP2 v. 1.1.4 with default settings [84] to predict the novel miRNA. Two indexes, one for the genome and the other for the non-miRNA, were built to filter out noisy sequences from the ncRNA fragments in miRDP2 [84]. The number of clean small RNA reads was counted using Samtools idxstats and was input into the DESeq2 program in the R package to identify the differentially expressed miRNAs (if |log2(fold change)| > 1, padj < 0.05) [78,80]. The DEMs between each time point after inoculation vs. 0 hai (the control with mock inoculation) were further identified in each line. DEM2Ls were also defined in the same way as DEG2Ls. MiRanda v1.9 with default settings [85,86,87,88] was used to predict the target genes of the DEMs in the *S. officinarum* genome, and these were annotated in the same way as for mRNA.

### 4.8. Weighted Correlation Network Analysis (WGCNA)

The read counts of each gene at all time points (0, 3, 6, 12, and 24 hai) of the two lines (540 and 664) were collected, and the genes with reading counts > 30 that were present in more than 50% of the samples (including mRNA, miRNA, and lncRNA) were kept and combined into one dataset. According to the read counts normalized by DEseq2 [80], the normalized dataset was used for WGCNA analysis using the WGCNA software package in R (v.1.73) [89]. A power function of 0.85 was selected to determine the soft power to generate an adjacency matrix. Modules were integrated if the distance between them was less than 0.25. Genes with an absolute value of |KME| over 0.9 were defined as hub genes.

The sub-networks were made by taking non-redundant genes with the strongest interactions and then taking the strongest interactions for each of these (regardless of redundancy) and graphing them in Cytoscape (v.3.10.3.).

### 4.9. SNP Identification

The “.sam” files generated from the Hisat2 alignment were combined according to each line: 540 and 664. SNP calling was conducted by BCFtools (-mpileup) to obtain “.vcf files” for both lines [90,91]. VCFtools v0.1.16 was then used to filter SNPs (--minQ 30 --min-alleles 2 --max-alleles 12 --max-missing 1 --remove-indels --recode --recode-INFO-all) and call SNPs on different chromosomes between two lines [92].

### 4.10. Metabolomic Analysis

The plant materials used for the metabolomic analysis were grown in the UF greenhouse, and the metabolomic analysis was conducted when the plants were two months old. The plants were cultivated in soil, with each plant individually placed in a separate pot. Two batches of seedlings were planted for metabolomic analysis: the first batch of seedlings was used to tell the time span from inoculation to symptom show up, and the second batch of seedlings was used for metabolomic analysis. After inoculation, when yellow spot lesions started to show up, orange rust-infected leaf sections (~4–5 cm long) of five sugarcane seedlings, with five biological replicates of each line from the second batch of seedlings, were collected and immediately frozen in liquid nitrogen and stored at −80 °C until ready for metabolomic analysis at the laboratory of UF Southeast Center for Integrated Metabolomics (SECIM). Each line had 5 independent samples as replicates and a pool of 5 samples for the metabolomic analysis. Sample preparation followed the projects carried out and analyzed in SECIM. Global metabolomics profiling was conducted using a Thermo Q-Exactive Oribtrap mass spectrometer with Dionex UHPLC and an autosampler. All samples were subjected to analysis under both positive and negative heated electrospray ionization, with a mass resolution of 35,000 at *m*/*z* 200 and separate injections. Based on an ACE 18-pfp (100 × 2.1 mm, 2 µm column), with mobile phase A as 0.1% formic acid in water and mobile phase B as acetonitrile, separation was obtained. While this polar-embedded stationary phase provides comprehensive coverage, it presents certain limitations in the coverage of very polar species. The flow rate was maintained at 350 µL/min and the column temperature was kept constant at 25 °C. About 4 µL was injected for negative ions and 2 µL for positive ions. MZmine was utilized to identify features, deisotope the data, align features, and conduct gap filling to capture any features potentially missed by the initial alignment algorithm [93]. The data were cross-referenced with the internal retention time metabolite library, sum-normalized, log-transformed, and autoscaled for PCA, PLS-DA, and significant metabolite identification. Student’s *t*-test was used for statistical analysis with a cutoff of 0.05.

Statistical analysis was performed separately on the positive and negative ion data. Univariate analysis was performed on all data sets. The numbers of significant metabolites (with *p*-values < 0.05 and |log2(fold change)| > 1) and known top metabolites were reported. By comparing the metabolomic profiles of non-inoculated plants and inoculated plants, potential metabolites originating from the pathogen can be excluded.

### 4.11. Transcriptional and Metabolomic Joint Analysis

The IDs of the DEGs and DEG2L homologous genes from resistant lines in rice that differentially produced metabolites in resistant line 540 were imported into metaboanalyst v6.0 (https://www.metaboanalyst.ca/) (accessed on 24 March 2024), using rice as the background to identify key pathways in the resistant line 540.

## 5. Conclusions

The results showed that the resistant line (540) had a smaller stomatal size and density. The transcriptomes of the two sister lines with contrasting resistance to sugarcane orange rust were compared at five time points after inoculation. At the five time points, a total of 9406 non-redundant DEG2Ls, 21 DEMs, and 701 DE-lncRNAs were identified. Networking analysis revealed that the turquoise and blue modules (4 miRNAs, 137 lncNRAs, and 5370 mRNAs) had the highest coefficient variation with hub genes (1506 miRNAs, 236 lncRNAs, and 7845 mRNAs). Finally, 21 miRNA families and 5 mRNAs were identified as quick-response genes with a high potential to confer resistance to early sugarcane orange rust infection. Moreover, 19 metabolites were identified in the resistant line through metabolomics. The transcriptomic enrichment analysis showed that line 540 has increased cell surface modification activity, suggesting possible increased surface receptors. DEG and coexpression analyses also revealed key genes involved in the biosynthesis of anti-fungal molecules, such as hordatines, arabidopyrones, and alkaloids. They also showed a substantial increase in lncRNA expression, playing a role in transcriptional regulation. Transcriptomic–metabolomic joint analysis suggested that the biosynthesis of phenylpropanoid derivatives with purported antioxidant and anti-fungal capabilities increased in line 540, especially those deriving from ferulate. This research provides lists of candidate genes, metabolites, morphological traits, and SNPs, which can be used as a reference for the future fine mapping of sugarcane orange rust disease resistance.

## Figures and Tables

**Table 1:** Induced metabolites in the 2 lines.

Induced Metabolite	Row Retention Time-Row *m*/*z*	Positive/Negative Mode	Line
2,3-dihydroxyisovalerate	133.0507-1.38	Negative Mode	540
3-4-hydroxyphenylpyruvate	163.0387-7.52	Positive Mode	540
3-hydroxyphenylacetate	151.0400-8.30	Negative Mode	540
4-hydroxyphenylacetate/mandelic acid	151.0400-7.11	Negative Mode	540
6-deoxy-l-galactose	163.0611-0.96	Negative Mode	540
allantoin	157.0367-0.81	Negative Mode	540
	193.0135-0.80	Negative Mode	540
arabinose	195.0509-0.73	Negative Mode	540
D—galacturonic acid	175.0236-0.89	Negative Mode	540
D-glyceric acid	233.0295-0.90	Negative Mode	540
D—raffinose	503.1617-0.93	Negative Mode	540
	549.1674-0.96	Negative Mode	540
ferulate	193.0500-9.32	Negative Mode	540
glucose/fructose	215.0328-0.74	Negative Mode	540
	217.0296-0.74	Negative Mode	540
ll-2,6-diaminoheptanedioate	189.0881-0.83	Negative Mode	540
orotate	155.0099-1.25	Negative Mode	540
phenethylamine	105.0702-7.23	Positive Mode	540
theobromine	181.0729-7.00	Positive Mode	540
uracil	111.0199-1.25	Negative Mode	540
11-dehydro-2,3-dinor TXB2	339.1809-10.33	Negative Mode	664
3-hydroxy-3-methylglutarate	161.0455-3.13	Negative Mode	664
	183.0274-3.15	Negative Mode	664
	185.0418-3.10	Positive Mode	664
betaine	152.0481-0.78	Negative Mode	664
l-arginine	209.0800-0.74	Negative Mode	664
2-acetamido-2-deoxy-beta-D-glucosylamine	203.1025-0.88	Positive Mode	540 664
2-Hydroxyhippuric acid	194.0459-7.57	Negative Mode	540 664
caffeate	163.0388-8.31	Positive Mode	540 664
catechol	109.0291-7.24	Negative Mode	540 664
phenylpyruvate	209.0454-8.42	Negative Mode	540 664
	209.0459-8.33	Negative Mode	540 664
pyridoxine	170.0808-2.94	Positive Mode	540 664
shikimate	219.0510-0.95	Negative Mode	664
	219.0510-1.13	Negative Mode	540 664
	219.0510-1.20	Negative Mode	540 664

## Data Availability

The original data presented in the study are available in BioProject ID PRJNA1196418 (“RNAseq profiling of sugarcane sister lines with opposite resistance to Orange Rust disease”) at https://www.ncbi.nlm.nih.gov/bioproject/PRJNA1196418/.

## Supplementary Materials

The following supporting information can be downloaded at: https://www.mdpi.com/article/10.3390/ijms26083490/s1.
