# Supplementary material for: Exploring Morphological, Transcriptomic, and Metabolomic Differences Between Two Sister Lines with Contrasting Resistance to Orange Rust Disease in Sugarcane
Source: Int J Mol Sci. 2025 Apr 8;26(8):3490. doi: 10.3390/ijms26083490 (PMC12027349; doi:10.3390/ijms26083490)
Supplement: Supplementary file 1 [file ijms-26-03490-s001.zip › ijms-3543364-Suplementary Figures.pdf]

Supplemental figures

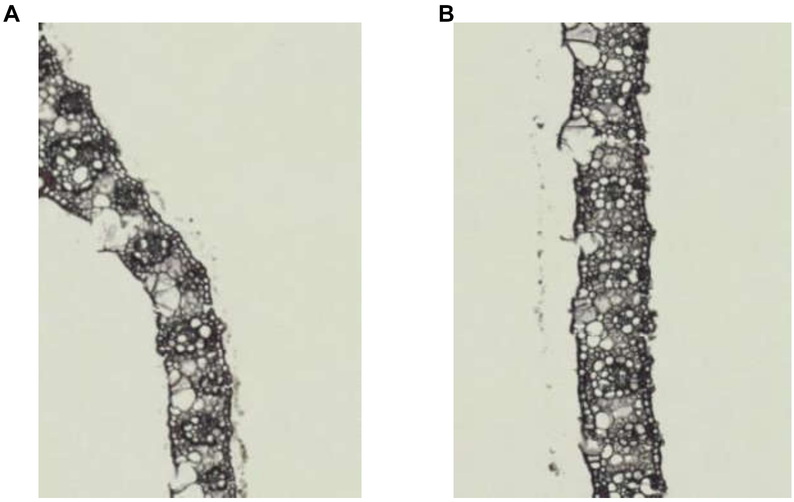

Figure S1: The cross sections of the (A) leaves of the line 540 and (B) leaves of the line 664.

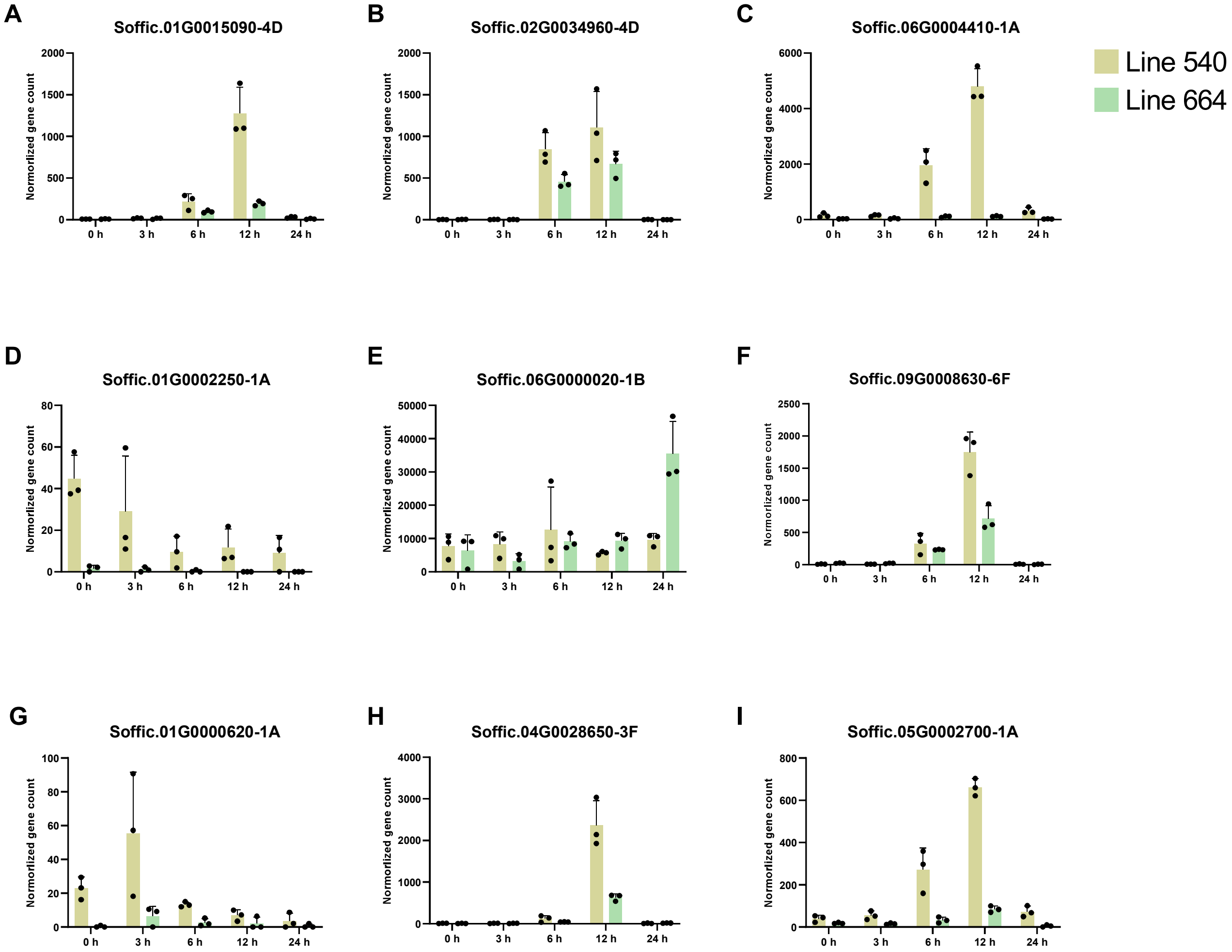

Figure S2: Normalized counts of certain DEG2L genes.

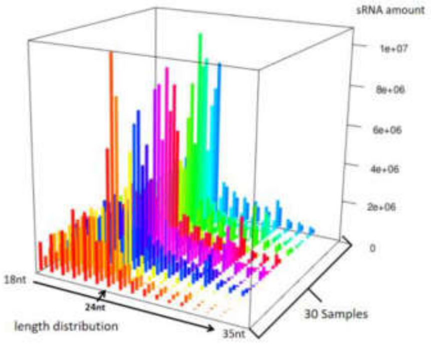

Figure S3: sRNA Length distribution.

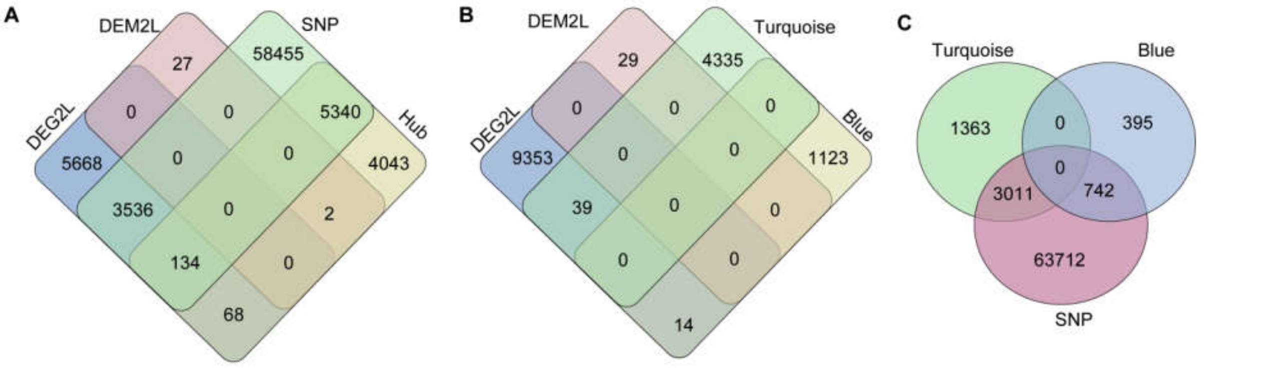

Figure S4: (A) The Venn diagram of the DEG2L DEM2L, hub genes, and genes with SNPs. (B) The Venn diagram of the DEG2L, DEM2L, and hub genes in the turquoise and blue module. (C) The Venn diagram of the hub genes in the turquoise and blue module and genes with SNPs.

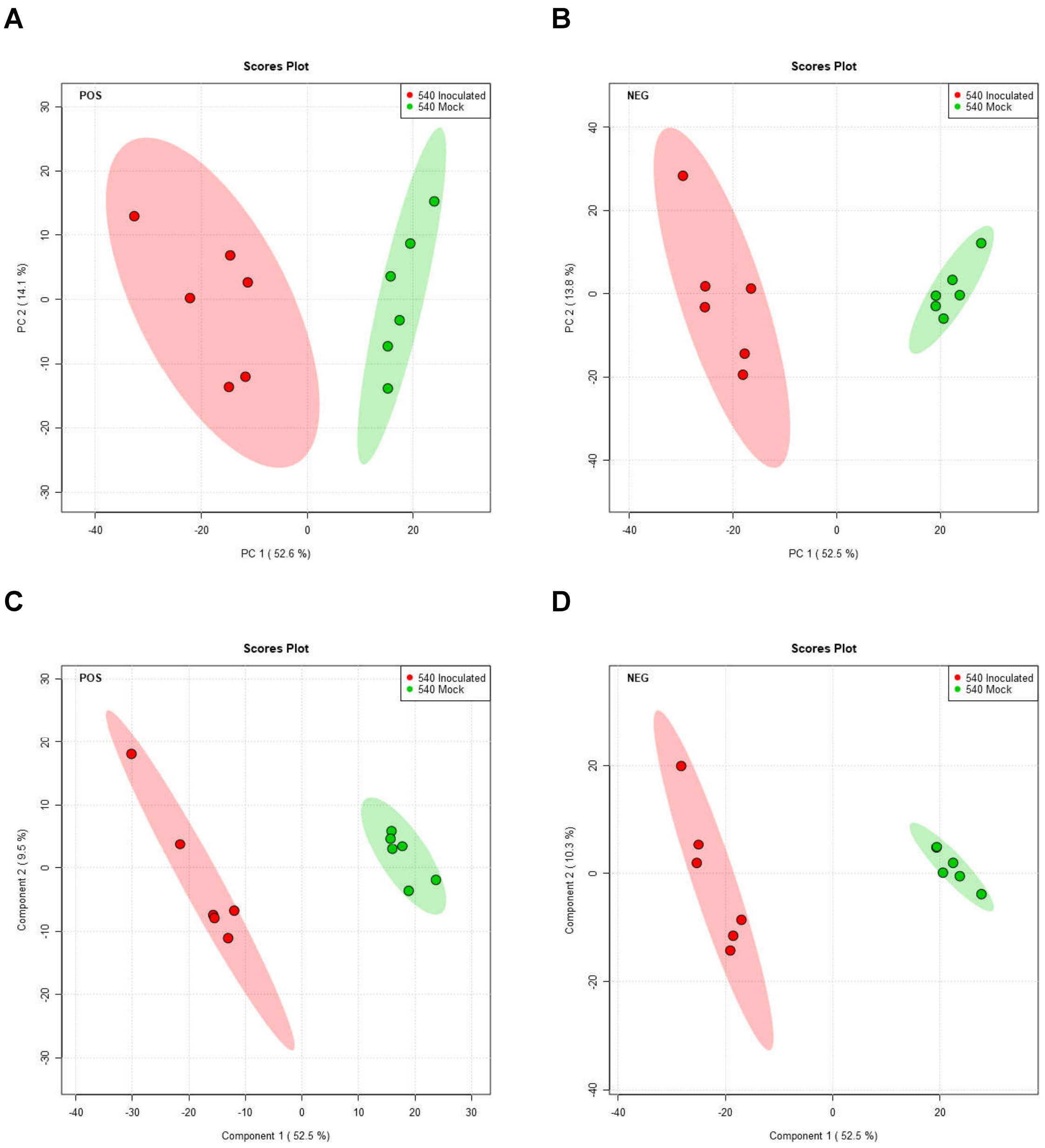

Figure S5: PCA of the (A) positive and (B) negative data set of the resistant line 540. PLS-DA of the (C) positive and (D) negative mode data set of the line 540.

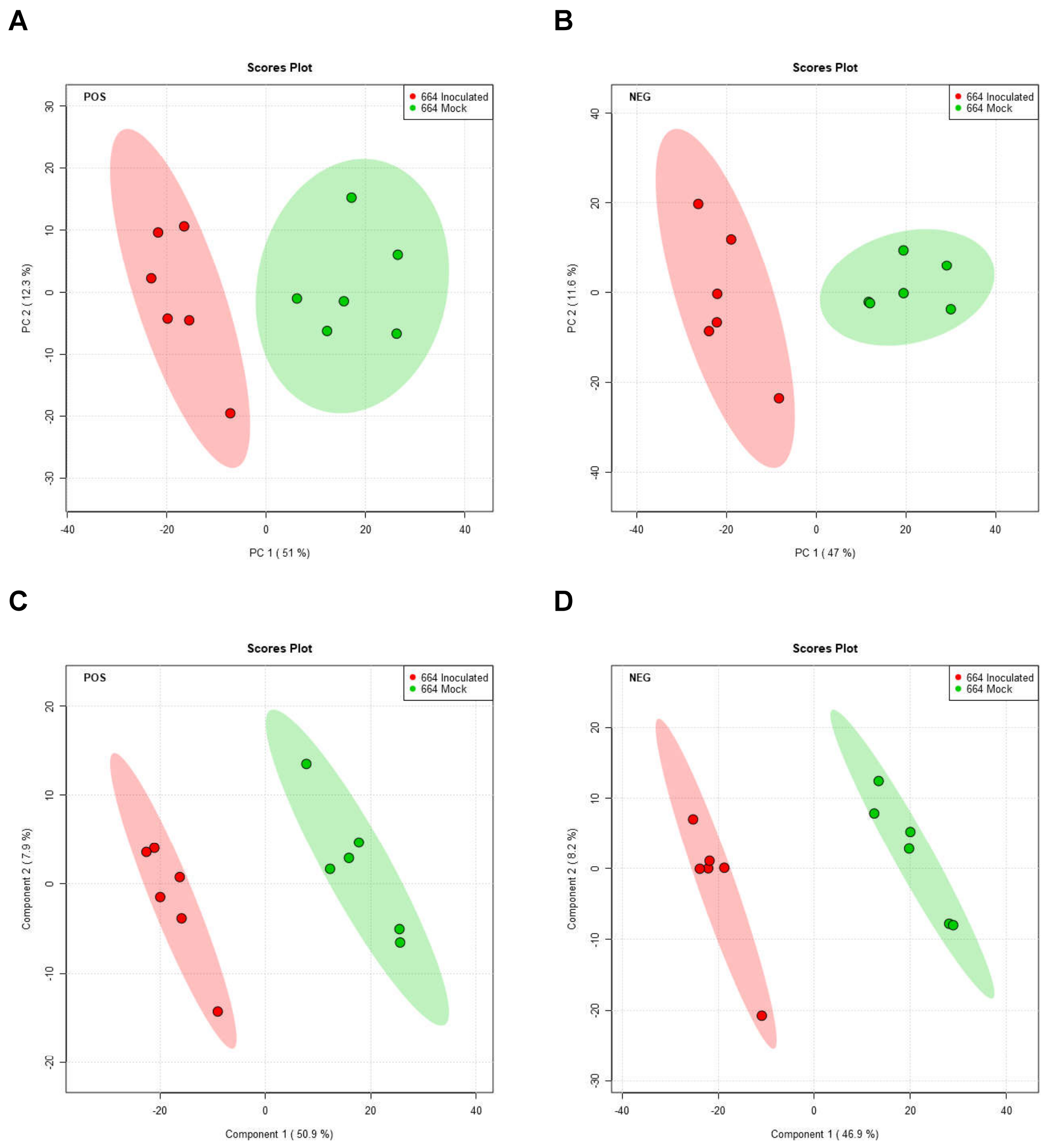

Figure S6: PCA of the (A) positive and (B) negative data set of the line 664. PLS-DA of the (C) positive and (D) negative mode data set of the susceptible - line 664.

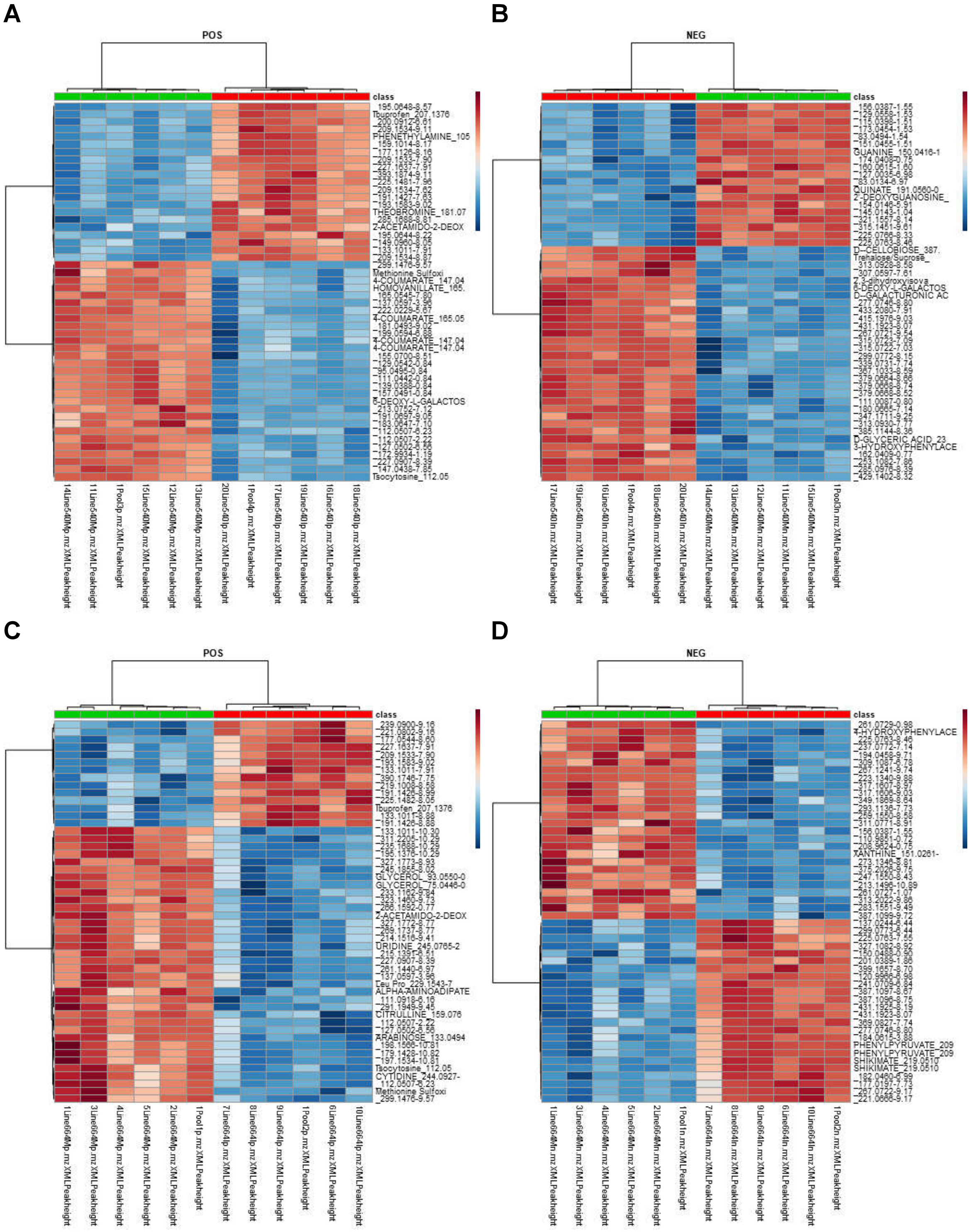

Figure S7 Heat maps of the top 50 from the (A) positive mode and (B) negative mode in the resistant - line 540. And Heat maps of the top 50 from the (C) positive mode and (D) negative mode in the susceptible - line 664.
